# A modular synthetic biology toolkit unlocks metabolic engineering of the industrially relevant alga *Nannochloropsis*

**DOI:** 10.1007/s44307-026-00096-w

**Published:** 2026-02-03

**Authors:** Yutan Guo, Zhixiong Li, Hao Wang, Jie Zheng, Zhiwei Liang, Han Sun, Wenguang Zhou, Jin Liu

**Affiliations:** Key Laboratory of Poyang Lake Environment and Resource Utilization Ministry of Education, Engineering Research Center of Watershed Carbon Neutrality of Ministry of Education, Center for Algae Innovation & Engineering Research, School of Life Sciences and School of Resources and Environment, Nanchang University, Nanchang, 330031 China

**Keywords:** Algal biotechnology, Carotenoid biosynthesis, Green production, Metabolic engineering, Modular assembly, Synthetic biology

## Abstract

*Nannochloropsis* is an industrially relevant marine microalga with exceptional potential as a chassis for sunlight-driven CO_2_ valorization. However, its broad application in synthetic biology has been constrained by the lack of a standardized and modular genetic toolbox. Here, we report the development of a comprehensive Modular Cloning (MoClo) toolkit for *Nannochloropsis*, based on Golden Gate assembly and a standard syntax. The toolkit comprises 91 domesticated genetic parts spanning promoters, signal peptides, selectable markers, reporter genes, tags and terminators. A large subset of these parts, including several not previously evaluated in *Nannochloropsis*, was functionally validated, enabling convenient and reliable transformant selection, immunodetection, and subcellular localization. To demonstrate the utility of the toolkit for multi-gene pathway engineering, modularly assembled keto-carotenoid biosynthetic pathways were introduced into *Nannochloropsis*, leading to substantial accumulation of canthaxanthin (4.5 mg g^−1^) or astaxanthin (2.8 mg g^−1^). Collectively, this flexible and expandable MoClo toolkit establishes a standardized foundation for synthetic biology in *Nannochloropsis*, enables rapid design-build-test cycles for multi-gene constructs, and advances the use of industrial microalga for sustainable, CO_2_-based production of value-added biochemicals.

**Supplementary Information:**

The online version contains supplementary material available at 10.1007/s44307-026-00096-w.

## Introduction

Our ecosystem faces grave environmental challenges due to the relentless rise in atmospheric carbon dioxide levels. In the quest for carbon neutrality, bio-sequestration has emerged as an eco-friendly and sustainable solution (Zhou et al. 2017). Within this approach, algae, particularly microalgae, are considered as crucial players in mitigating the carbon dioxide problem, due to the advantageous characteristics such as exceptional photosynthetic capabilities, rapid growth, strong resilience to environmental conditions, and ability to thrive without occupying arable lands (Daneshvar et al. 2022). Besides producing oils for biofuel production, microalgae are able to convert the photosynthetically fixed carbon dioxide (CO_2_) to a variety of value-added biochemical compounds, and are thus considered as promising sun light-driven cell factories for CO_2_ valorization and green production (Xu et al. 2024; Kumar et al. 2020).

Advances in genetic tools and genome editing techniques have significantly expanded the potential applications of algae, enabling the genetic manipulation of various species for photoautotrophic biochemical production (Jeong et al. 2023; Fayyaz et al. 2020; Sproles et al. 2021). Notable examples include the model green alga *Chlamydomonas reinhardtii*, the model diatom *Phaeodactylum tricornutum*, and the heterokont alga *Nannochloropsis oceanica*. The latter, *N. oceanica*, is a unicellular marine species of industrial relevance, known for its ability to accumulate high levels of triacylglycerol (Xu 2022). Rich in eicosapentaenoic acid (EPA), a high-value ω3 long-chain polyunsaturated fatty acid, *N. oceanica* has gained increasing attention as a new food resource for human consumption (Ye et al. 2024). *N. oceanica* is a haploid alga and its nuclear genome has long been fully sequenced and extensively annotated (Vieler et al. 2012; Wang et al. 2014). Besides, genetic tools for gene overexpression, suppression, subcellular localization, and genome editing have been developed, with demonstrated stability in transgene expression (Wei et al. 2017b; Poliner et al. 2018a, b; Wang et al. 2021). These attributes have established *N. oceanica* as an promising photoautotrophic algal host for the synthesis and production of various bio-compounds via metabolic engineering approaches, including enhanced triacylglycerol storage (Wei et al. 2017a; Li et al. 2019; Zienkiewicz et al. 2017), increased EPA content and EPA abundance in triacylglycerol (Liu et al. 2022a; Xin et al. 2017; Shi et al. 2021; Zheng et al. 2025), and biosynthesis of keto-carotenoids like canthaxanthin and astaxanthin (Liu et al. 2024a, b; Canini et al. 2024). However, these efforts typically involve the introduction of no more than three transgenes and the procedures for plasmid construction remain time-consuming. The lack of a standardized, modular molecular toolbox for *N. oceanica* currently limits its utility as a light-driven algal chassis for synthetic biology applications. This limitation can be addressed through the implementation of Modular Cloning (MoClo) technology, which would enhance the organism’s utility in such applications.

MoClo, based on the Golden Gate cloning technique, is a standardized DNA assembly method that enables efficient and flexible construction of complex genetic circuits and multi-gene constructs (Weber et al. 2011). By leveraging Type IIS restriction enzymes that recognize asymmetric sequences and generate specific overhangs, MoClo facilitates seamless, one-pot assembly of multiple DNA fragments (Marillonnet and Grützner 2020). Since its inception, MoClo has been utilized to develop tailored toolkits for various model organisms, including the bacteria *Escherichia coli* (Lai et al. 2018; Nielsen et al. 2025), the yeast *Saccharomyces cerevisiae* (Lee et al. 2015; Guo et al. 2015), and the land plant *Nicotiana benthamiana* (Engler et al. 2014). Additionally, MoClo-based toolkits have been established for microalgae such as *Synechocystis* sp. and *Synechococcus elongatus* (Vasudevan et al. 2019) and *Chlamydomonas reinhardtii* (Crozet et al. 2018). These applications demonstrate that MoClo is a highly efficient tool for constructing gene expression modules, optimizing metabolic pathways, and engineering complex genetic networks.

In this study, we aimed to address the lack of a modular toolkit for *N. oceanica* by establishing a MoClo toolkit comprising 91 genetic parts including promoters, terminators, selectable markers, fluorescent reporters and tags. These parts were assembled into vectors harboring one, two or three gene expression cassettes for functional validation in *N. oceanica*. We observed that commonly used promoters such as *P*_*RIBI*_ (from ribosomal subunit), *P*_*VCP2*_ (from violaxanthin/chlorophyll *a* binding protein 2), and *P*_*TUB*_ (from β-tubulin) showed distinctive activities when paired with different terminators. Three fluorescent proteins (eGFP, YFP, and StayGold) were assembled and validated for fluorescence detection and facile subcellular co-localization in the chloroplast and nucleus. We also evaluated the utility of five affinity tags for immunodetection. Furthermore, we assembled and validated canthaxanthin and astaxanthin biosynthesis modules in *N. oceanica*. Our findings provide a flexible and expandable MoClo toolkit that enables rapid design and assembly of multi-gene constructs, significantly reducing the complexity and cost of genetic engineering. The modular design will streamline metabolic pathway optimization and facilitate synthetic biology applications in the industrially relevant *Nannochcloropsis* species for transforming CO_2_ to various value-added products.

## Results and discussion

### Design of the *Nannochloropsis* MoClo toolkit

**Fig. 1:**
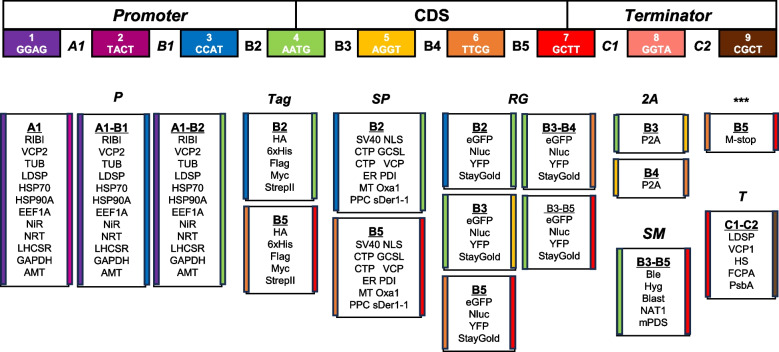
Schematic representation of modular genetic elements for *Nannochloropsis*. This figure employs the Plant MoClo Syntax color-coding scheme to indicate fusion sites. A1, B1-B5, C1 and C2 designate the positions where genetic elements can be assembled. All components of the *Nannochloropsis* MoClo toolkit are primarily categorized based on their functions, including promoters (P), immuno and purification tags (Tag), selectable markers (SM), signal peptides (SP), reporter genes (RG), 2A peptide, multi-stop (***), and terminators (T). The colored stripes on the left and right sides of each module represent the 5' and 3' fusion sites, respectively, following the color code indicated at the top to ensure compatibility in modular assembly. RIBI, ribosomal bifunctional; VCP, violaxanthin/chlorophyll *a*-binding protein; TUB, β-tubulin; LDSP, lipid droplet surface; HSP70, heat shock protein 70; HSP90A, heat shock protein 90A; EF1A, elongation factor 1A; NiR, nitrite reductase; NRT, nitrate reductase; LHCSR, light-harvesting complex stress-related; GAPDH, glyceraldehyde-3-phosphate dehydrogenase; AMT, ammonium transporter; SV40 NLS, nuclear localization sequence of SV40; CTP GCSL, chloroplast transit peptide of glycine cleavage system L protein; CTP VCP, chloroplast transit peptide of violaxanthin/chlorophyll* a* binding protein; ER PDI, ER-targeting peptide of protein disulphide isomerase; MT Oxa1, mitochondria‐targeting peptide of oxidase assembly protein 1; PPC sDer1-1, periplastidal compartment-targeting peptide of symbiont degradation in the ER 1; eGFP, enhance green fluorescence protein; Nflu, nano luciferase; YFP, yellow fluorescent protein; StayGold, a GFP variant; P2A, 2A peptide; Ble, zeocin resistant gene; Hyg, hygromin B resistance gene; Blast, blasticidin S resistance gene; NAT1, nourseothricin resistance gene; mPDS, modified phytoene desaturase gene; HS, heat shock protein; FCPA, fucoxanthin-chlorophyll-protein *a*; PsbA, photosystem II protein D1. See Dataset S1 for the detailed information of genetic parts

The modular design approach has proven to be highly efficient in synthetic biology, where standardized workflows enable the flexible assembly of genetic parts into various functional modules (Weber et al. 2011). To build the *Nannochloropsis* MoClo toolkit, we adopted the syntax proposed by the plant synthetic biology community (Patron et al. 2015). This toolkit included level 0 vectors with eight distinct fusion sites for cloning, designed to accommodate standard genetic parts. In total, the MoClo toolkit consists of 91 parts representing 39 unique genetic elements positions at various sites within the standard framework, enabling flexible modular assembly (Fig. 1 and Table 1). The sequence information for these genetic elements was provided in Dataset S1. The genetic elements in the *Nannochloropsis* MoClo toolkit were grouped into several functional categories. These include 12 promoters (P), distributed across three different positions to allow efficient regulation of gene expression; five tags (Tag), located at either the N-terminus or C-terminus of the target gene to facilitate protein detection and purification; five selectable markers (SM) for enabling transformation with multiple vectors; six signal peptides (SP), each offering two position options for subcellular targeting of proteins; four reporter genes (RG), with five position options for versatile gene function monitoring; one 2A peptide (2A) for protein fusion expression; one polygenic terminator (***) and five terminators (T) (Table 1). Genetic parts on level 0 vectors, through a standardized assembly process, could be integrated into level 1 vectors to form modules with specific functions. The modules on level 1 vectors could be further assembled into level 2 vectors to create devices with multiple expression cassettes following predefined design rules (Fig. S1).

**Table 1:** Unique and total genetic parts available in the *Nannochloropsis* MoClo toolkit

Type of parts	Abbreviation	Unique parts	Total parts
Promoters	P	12	36
Tags	T	5	10
Selectable markers	SM	5	5
Signal peptides	SP	6	12
Reporter genes	RG	4	20
2A peptide	2A	1	2
Multi-Stop	***	1	1
Terminators	T	5	5
Total		39	91

***means the multi-stop codon sequences

### Modular assembly and evaluation of selectable marker

**Fig. 2:**
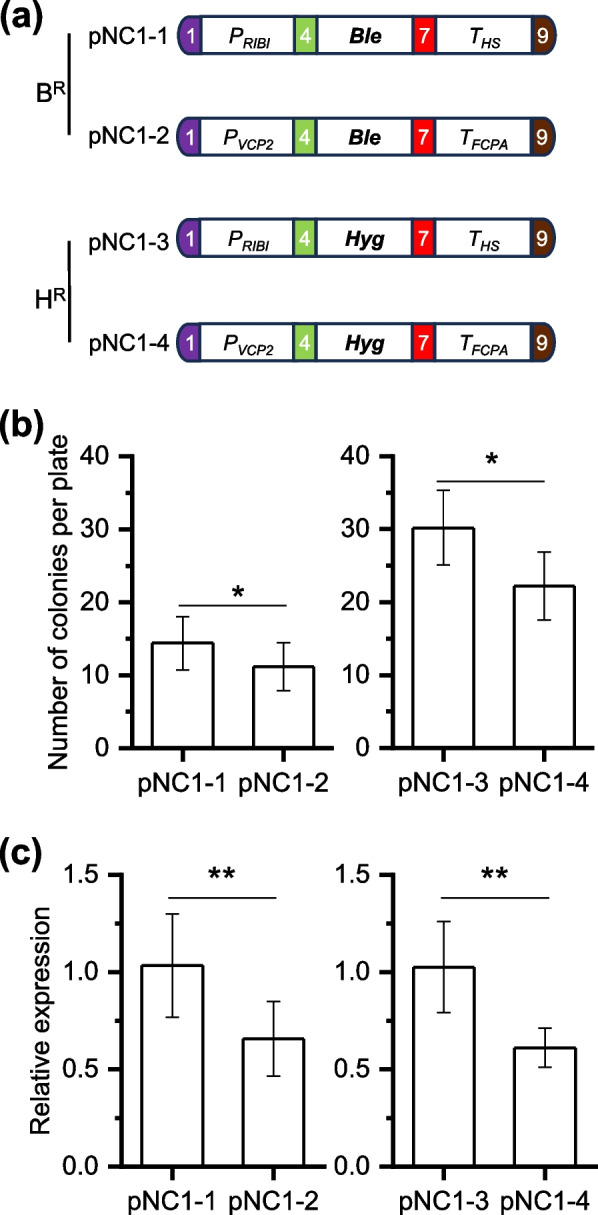
Modular assembly and validation of selectable marker genes. **a** Schematic illustration of the four constructs containing either the Ble (Bleomycin resistance) or Hyg (Hygromycin resistance) genes under the control of different promoter-terminator combinations: *P*_*RIBI*_-*Ble*-*T*_*HS*_ (pNC1-1), *P*_*VCP2*_-*Ble*-*T*_*FCPA*_ (pNC1-2), *P*_*RIBI*_-*Hyg*-*T*_*HS*_ (pNC1-3), and *P*_*VCP2*_-*Hyg*-*T*_*FCPA*_ (pNC1-4). **b** Number of resistant algal colonies per plate for each construct. Colonies were counted after antibiotic selection. **c** Transcriptional expression of *Ble* gene in pNC1-1 and pNC1-2 transformants (left), and of *Hyg* gene in pNC1-3 and pNC1-4 transformants (right). Data represent mean ± SD (*n* = 5 in **b** and *n* = 6 in **c**). Asterisks indicate statistical significance determined Student’s *t*-test (* *p* < 0.05, ** *p* < 0.01)

Selectable markers are crucial for selection of algal transformation. Five selectable markers are included in the toolkit (Fig. 1), which have been shown to function effectively in *Nannochloropsis*. These marker genes are the zeocin resistant gene *Ble* from *Streptoalloteichus hindustanus* (Kilian et al. 2011), the hygromycin B resistant gene *Hyg* from *Streptomyces hygroscopicus* (Vieler et al. 2012), the blasticidin resistant gene *Blast* from *Aspergillus terreus* and the nourseothricin resistant gene *NAT1* from *Streptomyces noursei* (Poliner et al. 2020), and the modified phytoene desaturase gene *mPDS* from *N. oceanica* that confers resistance to norflurazon (Liu et al. 2022b). For convenience, we here specifically evaluated *Ble* and *Hyg*, both driven by the well-characterized and commonly used promoter-terminator pair *P*_*RIBI*_-*T*_*HS*_ or *P*_*VCP2*_-*T*_*FCPA*_, resulting in four vectors pNC1-1 to pNC1-4 (Fig. 2a). The *RIBI* promoter (*P*_*RIBI*_) is derived from the intergenic region between two ribosomal subunits and the *HS* terminator (*T*_*HS*_) is from a heat shock protein-encoding gene of *N. oceanica* origin (Poliner et al. 2020), while the *VCP2* promoter is from violaxanthin/chlorophyll *a*-binding protein 2 gene of *N. oceanica* and the *FCPA* terminator is from fucoxanthin-chlorophyll-protein *a* gene of *Phaeodactylum tricornutum* origin (Wei et al. 2017a; Karas et al. 2015). The transformation efficiency of the four vectors for *N. oceanica* was assessed by counting the antibiotic resistant colonies and the results indicated that *P*_*RIBI*_-*T*_*HS*_ had greater performance than *P*_*VCP2*_-*T*_*FCPA*_ regardless of the selectable markers used (*Ble* or *Hyg*) (Fig. 2b). In line with the transformation efficiency, *P*_*RIBI*_-*T*_*HS*_ also drove higher transcriptional expression of the selectable marker genes compared to *P*_*VCP2*_-*T*_*FCPA*_, as determined by RT-qPCR analysis (Fig. 2c).

### Modular assembly and evaluation of promoter-terminator pairs

**Fig. 3:**
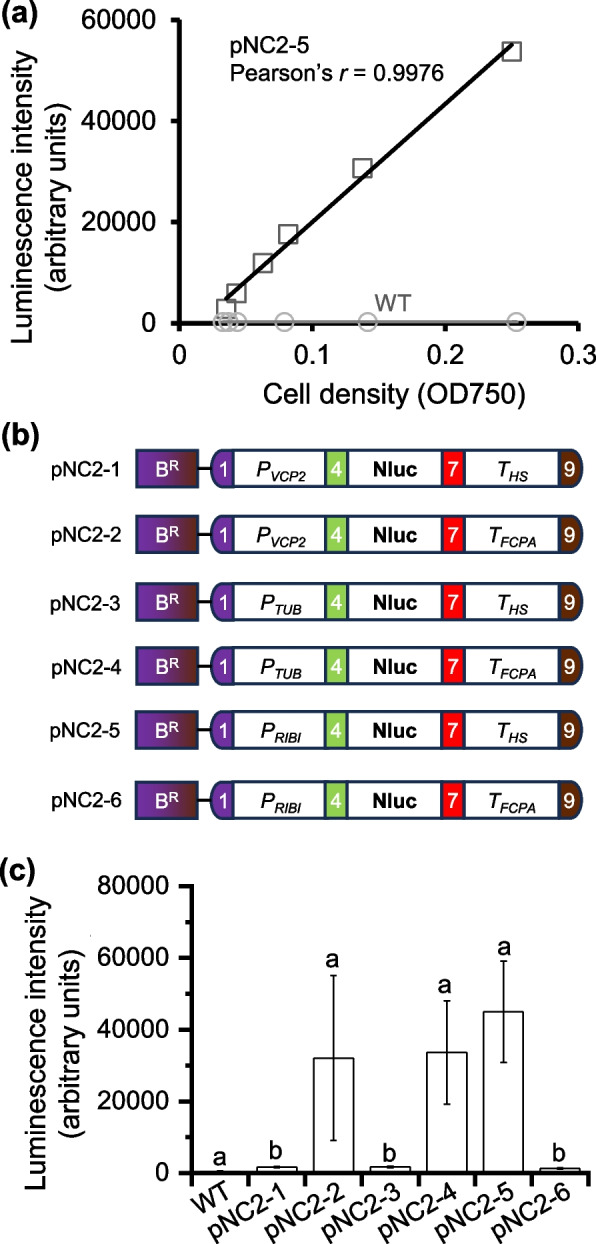
Modular assembly and validation of reporter gene expression driven by various promoters and promoter-terminator combinations. **a** Linear relationship between luminescence intensity and cell density (expressed as OD750) of *Nannochloropsis* strains. The plot displays the Pearson’s correlation coefficient (*r*). Circle, WT; square, pNC2-5. **b** Schematic illustration of the six constructs pNC2-1 to pNC2-6, each containing the NanoLuc (Nluc) gene under the control of different promoter-terminator combinations. **c** Comparison of luminescence intensity of WT and transformants with pNC2 constructs. Data represent mean ± SD; five individual transformants for each construct were used for determination. Different letters above the bars indicate the significant difference (*p* < 0.05), based on one-way analysis of variance (ANOVA)

The transformation assay indicated the difference of promoter-terminator pairs in driving transgene expression (Fig. 2). As a further step, NanoLuciferase (Nluc), which allows more sensitive and quantitative analysis of transgene expression in algae (Crozet et al. 2018; Poliner et al. 2020), was adopted as the reporter to evaluate the performance of promoter-terminator pairs. The wild type *N. oceanica* cells showed only a basal level of luminescence intensity; by contrast, the *N. oceanica* transformant with Nluc expression showed strong luminescence intensity, which exhibited a good linear correlation with the cell density (OD750, optical density at 750 nm) (Fig. 3a). Besides *P*_*RIBI*_ and *P*_*VCP2*_, the promoter from β-tubulin gene (*P*_*TUB*_) (Radakovits et al. 2012) was also evaluated, paired with either* T*_*HS*_ or *T*_*FCPA*_ to drive Nluc expression, which together with the *Ble* expression cassette were assembled into level 2 vectors (pNC2-1 to pNC2-6; Fig. 3b). Notably, the pNC2-5 transformants exhibited the highest luminescence intensity, followed by pNC2-4 and pNC2-2, with values within the linear range (Fig. 3a and c). By contrast, the pNC2-1, pNC2-3 and pNC2-6 transformants, although higher than WT, showed much lower luminescence intensity compared to pNC2-2, pNC2-4 and pNC2-5 transformants, respectively (Fig. 3c). Notably, pNC2-1 and pNC2-2 share the same promoter, but are paired with different terminators to drive Nluc expression; pNC2-3 and pNC2-4, as well as pNC2-5 and pNC2-6, follow the same pattern (Fig. 3b). These results suggest that the activity of the promoter in *N. oceanica* is impacted greatly by the paired terminator. Therefore, future research on evaluation and selection of promoter elements for *Nannochloropsis* synthetic biology should carefully consider the suitability of promoter and terminator pair. This phenomenon has also been observed in other algae such as Chlamydomonas (Crozet et al. 2018).

### Modular assembly and evaluation of fluorescent proteins for subcellular localization

**Fig. 4:**
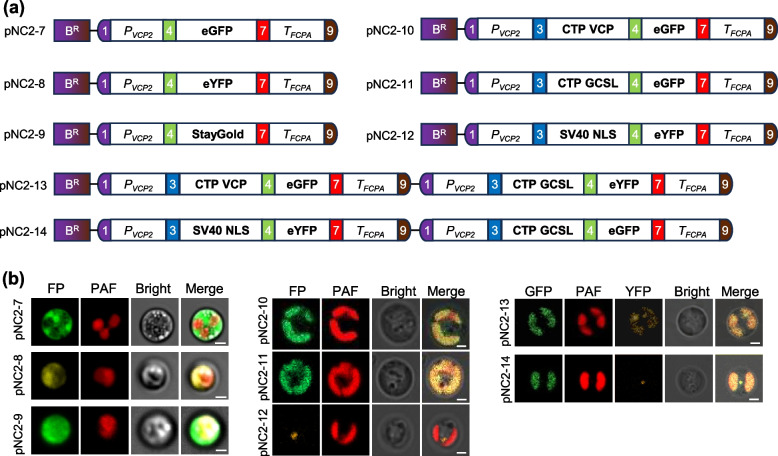
Modular assembly and validation of fluorescent proteins targeting to different subcellular compartments. **a** Schematic illustration of the constructs harboring fluorescent protein-coding genes controlled by the VCP2 promoter and the FCPA terminator. Plasmids pNC2-7, pNC2-8, and pNC2-9 were constructed to express eGFP, eYFP, and StayGold, respectively; pNC2-10 and pNC2-11 were assembled with chloroplast transit peptides (CTP VCP and CTP GCSL, respectively) for directing eGFP to the chloroplast, while pNC2-12 was assembled with the SV40 nuclear localization seqeunce (NLS) for nuclear targeting of YFP; pNC2-13 was assembled for simultaneous chloroplast targeting of eGFP and YFP, whereas pNC2-14 was for nuclear targeting of YFP and chloroplast targeting of eGFP. **b** Fluorescence microscopy observation of subcellular localization of fluorescent proteins. FP, fluorescent protein signal; PAF, chlorophyll autofluorescence; Bright, bright-field imaging. Scale bar: 2 μm

Fluorescent protein tagging serves as a pivotal tool in cell biology for visualizing protein dynamics and subcellular compartmentalization. Here we systematically evaluated the modular utility of several fluorescent proteins in *N. oceanica*, including the enhanced green fluorescent protein (eGFP) (Wei et al. 2017a), enhanced yellow fluorescent protein (eYFP) (Kassaw et al. 2022), and StayGold, a highly photostable and bright variant of GFP (Hirano et al. 2022). The genes encoding these fluorescent proteins were initially assembled into level 1 vectors driven by *P*_*VCP2*_-*T*_*FCPA*_, and then into level 2 vectors with the *Ble* expression cassette, resulting in vectors pNC2-7 to pNC2-9, respectively (Fig. 4a). As expected, the transformants with pNC2-7 or pNC2-9 exhibited strong green fluorescence in the cytosol, and the pNC2-8 transformants showed strong yellow fluorescence in the cytosol (Fig. 4b), in line with the previously reported localization of fluorescent proteins when expressed alone in algae (Poliner et al. 2018a; Shi et al. 2021).

Implementation of *Nannochloropsis* synthetic biology may require the targeting of heterologous proteins to specific compartments for proper function, which can be achieved by fusing the proteins to signal peptides. A total of six validated transit peptides were included in the MoClo toolkit (Fig. 1): the well-used nuclear localization sequence from simian vacuolating virus 40 (SV40 NLS) (Poliner et al. 2018b), the chloroplast transit peptides from *N. oceanica* glycine cleavage system protein L (CTP GCSL) and violaxanthin/chlorophyll *a* binding protein 1 (CTP VCP) (Moog et al. 2015; Koh et al. 2019), the ER-targeting peptide from protein disulphide isomerase (ER PDI), the mitochondria-targeting peptide from oxidase assembly protein 1 (MT Oxa1) and the periplastidial compartment-targeting peptide from symbiont degradation in the ER 1 (PPC sDer1-1) (Moog et al. 2015). For convenience, we evaluated only CTP GCSL, CTP VCP, and SV40 NLS, which were assembled at the N-terminus of eGFP or eYFP (Fig. 4a, vectors pNC2-10 to pNC2-12). Clearly, the eGFP signal (green) was observed in the chloroplast of transformants with pNC2-10 or pNC2-11, while the eYFP signal (yellow) was confined to a single spot (Fig. 4b), similar to the previously reported localization pattern of NLS-directed GFP in *N. oceanica* (Poliner et al. 2018a) indicative of its targeting to nucleus. Besides, the eGFP and eYFP cassettes, together with the *Ble* cassette, were assembled into level 2 vectors to form pNC2-13 and pNC2-14 (Fig. 4b). Transformants with pNC2-13 (where eGFP and eYFP were directed by different CTPs) showed both eGFP and eYFP signals in the chloroplast; in contrast, transformants with pNC2-14 (where eGFP and eYFP were directed by CTP and NLS, respectively) exhibited eGFP signal in the chloroplast and eYFP signal in the nucleus (Fig. 4b; Fig. S2), demonstrating the utility of the system for co-localization studies in *N. oceanica*. It is worth noting that the signal peptides can be flexibly assembled at either the N- or C-terminus of fluorescent proteins or other proteins of interest according to the MoClo toolkit design (Fig. 1).

### Modular assembly and evaluation of multiple fusion tags for immuno- detection and purification

**Fig. 5:**
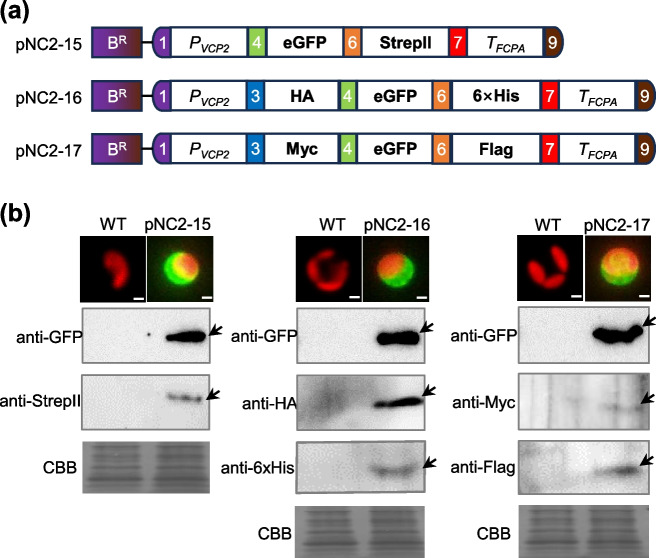
Assembly and validation of tagged protein constructs. **a** Schematic illustration of the constructs harboring eGFP-StrepII fusion (pNC2-15), HA-eGFP-6 × His fusion (pNC2-16), and Myc-eGFP-Flag fusion (pNC2-17), respectively. **b** Immunoblotting with antibodies against either eGFP (anti-eGFP) or the appropriate tag (anti-strepII, anti-HA, anti-6 × His, anti-Myc, and anti-Flag; indicated within each panel) using protein extracts from WT and the transformants with pNC2-15, pNC2-16 or pNC2-17. CBB, Coomassie Brilliant Blue staining (protein loading control). Above the Immunoblotting panels are the fluorescence microscopy pictures of algal cells (red, chlorophyll autofluorescence; green, GFP fluorescence signal)

Fusion tags are essential tools used to facilitate immuno- detection and purification of recombinant proteins. Our MoClo toolkit includes five widely used epitope tags: HA, 6 × His, Flag, Myc, and StrepII (Fig. 1). Among these, the HA tag has been functionally validated in *N. oceanica* (Poliner et al. 2018a, b). Here we took advantage of the fluorescence visibility of eGFP and tagged it with StrepII at N-terminus (pNC2-15), with HA and 6 × His at N- and C-terminus (pNC2-16), or with Myc and Flag at N- and C-terminus (pNC2-17) (Fig. 5a). For the transformants with either pNC2-15, pNC2-16, or pNC2-17, eGFP was clearly visualized within the algal cells under fluorescent microscopy and was detected by immunoblotting with the antibody against eGFP (Fig. 5b; Fig. S3). Besides the previously validated HA tag, the other four epitope tags were also successfully immunodetected using the corresponding antibodies, showing specific bands (Fig. 5b; Fig. S3). This demonstrates the utility of these tags and multi-tag systems in *N. oceanica*. Notably, the HA tag gave rise to stronger immuno-signal than other four tags (Fig. 5b), suggesting that it may be more effective and should be prioritized for immuno-detection and affinity applications in *N. oceanica*.

### Modular assembly and evaluation of keto-carotenoid biosynthesis pathways

**Fig. 6:**
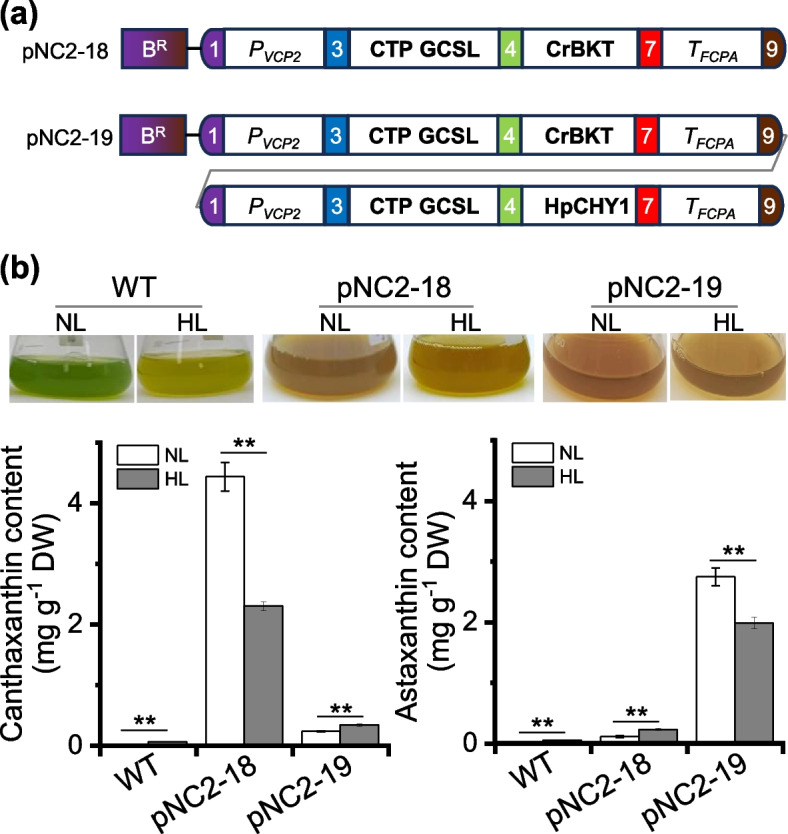
Assembly and validation of keto-carotenoid biosynthesis modules in *N. oceanica*. **a** Schematic illustration of the constructs harboring the canthaxanthin biosynthesis module (pNC2-18) and astaxanthin biosynthesis module (pNC2-19). **b** Quantification of canthaxanthin in pNC2-18 transformant and astaxanthin in pNC2-19 transformant under normal light (NL) and high light (HL) conditions. Data represent mean ± SD (*n* = 3). The culture pictures were shown above the column panel. Asterisks indicate statistical significance determined Student’s *t*-test (** *p* < 0.01)

One application of synthetic biology is introducing biosynthetic pathways for the production of target products in hosts. To evaluate the modular assembly and functionality of biosynthetic pathways using our *Nannochloropsis* MoClo toolkit, we selected the biosynthesis of two keto-carotenoids, canthaxanthin and astaxanthin. These keto-carotenoids are known for their strong antioxidant properties and having broad applications in food, feed, and nutraceuticals fields (Galasso et al. 2018; Rebelo et al. 2020). In microalgae such as *Haematococcus lacustris* and *Chromochloris zofingiensis*, astaxanthin biosynthesis from β-carotene involves two enzymes, a β-carotenoid ketolase (BKT) and a β-carotenoid hydroxylase (CHYB) (Zhang et al. 2021; Shah et al. 2016). For canthaxanthin biosynthesis, a β-carotenoid ketolase from Chlamydomonas (CrBKT), which efficiently ketolates β-carotene to form canthaxanthin (Zhong et al. 2011), was assembled with CTP GCSL at the N-terminus to create the level 2 vector pNC2-18 (Fig. 6a). For astaxanthin biosynthesis, CrBKT and a β-carotenoid hydroxylase from *H. lacustris* (HpCHYB), which efficiently hydroxylates canthaxanthin to produce astaxanthin (Liu et al. 2024a), were assembled as individual cassettes to form the level 2 vector pNC2-19, each tagged with CTP GCSL at its N-terminus (Fig. 6a).

The wild type (WT) *N. oceanica* cultures were green and turned to be slight yellow under high light (HL) conditions, with accumulation of extremely low canthaxanthin and astaxanthin (Fig. 6b). By contrast, transformants with pNC2-18 exhibited an orange color when cultured in flasks, and canthaxanthin content reached 4.5 mg g^−1^ dry weigh (Fig. 6b; Fig. S4). Similarly, transformants with pNC2-19 appeared as orange in flasks and accumulated astaxanthin, with a content of 2.8 mg g^−1^ dry weigh (Fig. 6b). The ketocarotenoid contents achieved through modular assembly here are comparable to those achieved using traditional cloning methods (Liu et al. 2024a, b). Differing from *H. lacustris* and *C. zofingiensis* that naturally synthesize keto-carotenoids but require stresses such as HL for inducing canthaxanthin/astaxanthin accumulation (Shah et al. 2016; Zhang et al. 2021), the *N. oceanica* transformants had higher canthaxanthin/astaxanthin contents under normal light (NL) compared to high light (HL) conditions (Fig. 6b). This is not surprising as the canthaxanthin or astaxanthin biosynthesis module introduced into *N. oceanica* was driven by the strong promoter *P*_*vcp2*_ (Fig. 6a). In *N. oceanica*, carotenoids, including β-carotene, the direct precursor for canthaxanthin and astaxanthin biosynthesis, are severely impaired by HL (Liu et al. 2023). In this context, the reduction of β-carotene under HL conditions may limit the substrate availability for CrBKT and HpCHYB, thereby restricting the biosynthesis of keto-carotenoids in the engineered *N. oceanica* strains. Considering the abundance of violaxanthin and astaxanthin (or canthaxanthin) (Fig. S4), these engineered *N. oceanica* strains may serve as sources of multiple carotenoids. Nevertheless, the astaxanthin content achieved in the engineered *N. oceanica* strains is still much lower than that in *H. lacustris*, which can reach up to 4% of cell dry weigh under stress conditions (Shah et al. 2016).

Astaxanthin has been reported to integrate into the thylakoid membranes in the astaxanthin-rich engineered *N. oceanica* strains (Liu et al. 2024a). In this study, we isolated thylakoid membranes from the canthaxanthin-rich engineered *N. oceanica* strain (pNC2-18 transformant) for carotenoid analysis. Notably, canthaxanthin was detected in the thylakoid membranes, constituting 4.4 mol per 100 mol of chlorophyll *a* (Table S1). Specifically, the violaxanthin–chlorophyll *a*-binding protein (VCP) contained 6.6 mol of canthaxanthin per 100 mol of chlorophyll *a*, while the photosystem I–light-harvesting complex (PSI-LHC) contained 3.4 mol of canthaxanthin per 100 mol of chlorophyll *a* (Table S1).

**Fig. 7:**
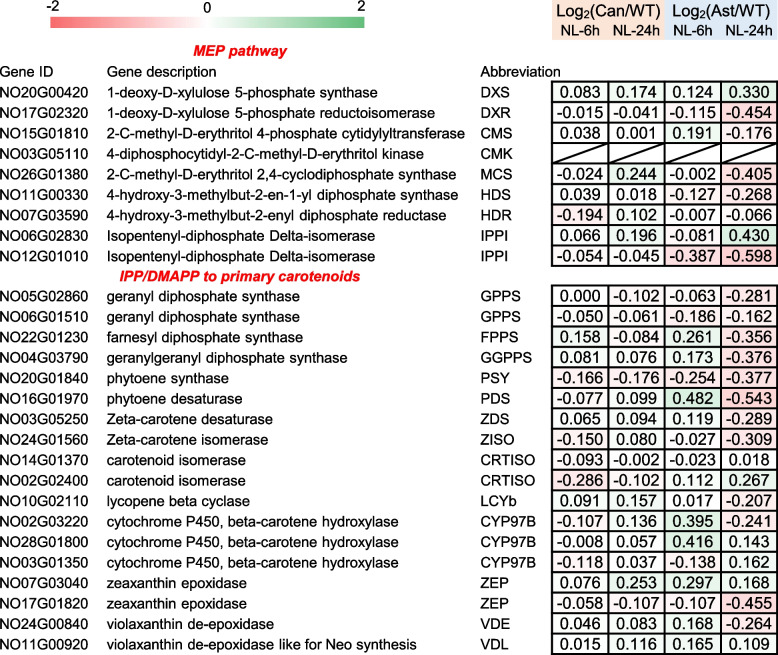
Transcript changes of genes involved in carotenogenic pathways. The data are expressed as log_2_ transformed fold change of genes in canthaxanthin-rich (Can) strain transformed with pNC2-18 or astaxanthin-rich (Ast) strain transformed with pNC2-19 compared to wild type (WT) *N. oceanica*

To understand whether the engineered keto-carotenoid accumulation impacts expression of endogenous carotenoid biosynthesis in *N. oceanica*, comparative transcriptomic analysis was conducted for WT, pNC2-18 (canthaxanthin-rich), and pNC2-19 (astaxanthin-rich) transformants based on the RNA-seq data at two time points under NL conditions (6 and 24 h; Fig. S5). In *N. oceanica*, the precursors for carotenoid biosynthesis, isopentenyl diphosphate (IPP) and dimethylallyl diphosphate (DMAPP), are derived from the 2-C-methylerythritol 4-phosphate (MEP) pathway located in the chloroplast (Liu et al. 2022b). Condensation of DMAPP and IPP molecules by GGPP synthase (GGPPS) produces GGPP, which is further condensed to phytoene by phytoene synthase (PSY) and then is converted to β-carotene by phytoene desaturase (PDS), ζ-carotene isomerase (ZISO), ζ-carotene desaturase (ZDS), carotenoid isomerase (CRTISO) and lycopene β-cyclase (LCYB). Hydroxylation of β-carotene by heme-containing cytochrome P450 enzymes (CYP97) gives rise to zeaxanthin, which can be interconverted to violaxanthin by zeaxanthin epoxidase (ZEP) and violaxanthin de-epoxidase (VDE). Clearly, the expression fold change of these genes in the carotenoid biosynthesis of engineered strains (canthaxanthin-rich versus WT and astaxanthin-rich versus WT) was less than 1.5 (Fig. 7), indicating that the engineered keto-carotenoid accumulation has only mild impact on the transcriptional expression of carotenoid biosynthesis. Future engineering efforts for further increasing ketocarotenoids in *N. oceanica* may lie in strengthening MEP pathway and β-carotene biosynthesis to provide precursors.

### Prospects of the established synthetic biology toolkit

To address the lack of standardized and modular molecular toolbox for advanced synthetic biology in the industrially relevant alga *Nannochloropsis*, we developed a MoClo toolkit comprising 91 domesticated genetic parts (Table 1), and validated many genetic parts of this toolkit for functionality in *N. oceanica*, including promoters, terminators, selectable markers, reporter genes, signal peptides, and tags that have been previously used in *Nannochloropsis* engineering (Figs. 2, 3, 4 and 5). Additionally, we demonstrated the efficacy of several components in *N. oceanica* for the first time, such as the fluorescent protein StayGold and the affinity tags 6 × His, Flag, Myc, and StrepII (Figs. 4 and 5). Furthermore, we successfully validated the biosynthetic pathways of value-added carotenoids canthaxanthin and astaxanthin in *N. oceanica* through modular assembly (Fig. 6). To test applicability of the MoClo toolkit in other *Nannochloropsis*, pNC2-18 was introduced into *N. gaditana*, and the resulting transformant produced substantial amount of canthaxanthin as well (Fig. S6). These findings indicate that our established MoClo toolkit works well for *N. oceanica* and *N. gaditana* and represents a major advance in *Nannochloropsis* synthetic biology. Due to the modular nature, this toolkit readily accommodates the integration of additional genetic parts, such as the dose-dependent fine-tuning regulatory elements with broad range activities (Kassaw et al. 2022), which can allow controllable expression of transgenes if they work in *Nannochloropsis*.

Thanks to the streamlined and versatile nature of the MoClo approach, genetic assembly is no longer a bottleneck for *Nannochloropsis* engineering. With the standardized components available in our toolkit, researchers can now design and construct complex constructs containing up to six distinct genes within a single level 2 vector in a matter of days. The availability of multiple selectable markers can enable transformation of *Nannochloropsis* with more than one level 2 vector, thus theoretically allowing the introduction of over ten genes. This capability will facilitate the reconstruction of complex pathways within the alga. Integration of our MoClo toolkit with the established technologies such as gene editing through CRISPR/Cas9 (Cas12) systems (Poliner et al. 2018b; Naduthodi et al. 2021) and high-throughput microfluidics (Yu et al. 2021) will enhance the utility of *Nannochloropsis* as a sunlight-driven photosynthetic chassis and support innovative synthetic biology approaches for both fundamental research and biotechnological applications.

## Materials and methods

### Algal strains and culture conditions

*Nannochloropsis oceanica* IMET1 (originally from the Institute of Marine and Environmental Technology, the University Systems of Maryland) was maintained on agar slants at 16 °C. The culture medium for the algal cultivation was F2 medium with minor modifications: 0.6 g L^−1^ NaNO_3_, 0.04 g L^−1^ NaH_2_PO_4_·H_2_O, 20 g L^−1^ sea salt (Wei et al. 2017a). When needed, the algae were recovered in the liquid modified F/2 medium (two-fold nitrogen and phosphorus nutrients of the original recipe and 20 g L^−1^ sea salt) under dim light for four days, and then inoculated into 50-mL flasks (containing 10 mL of the above liquid medium) under the following conditions: orbital shaking of 120 rpm, continuous illumination of 50 μmol photons m^−2^ s^−1^ and temperature of 23 °C. After six days of cultivation, the algal cells were then inoculated into 250-mL flasks containing 100 mL of liquid medium and cultured for four days under either 50 μmol photons m^−2^ s^−1^ (NL) or 300 μmol photons m^−2^ s^−1^ (HL).

### Design, molecular cloning, and assembly of genetic parts

Designs and analysis of DNA sequences were performed using SnapGene (v4.1.9, GSL Biotech LLC; Boston, USA). The coding sequences (CDS) of exogenous genes were codon-optimized based on the codon bias of *N. oceanica* and synthesized by Tsingke Biotechnology (China). DNA fragments of the genetic elements (e.g., promoters, terminators, selectable markers) were amplified using 2 × Phanta® Flash Master Mix (Dye Plus) (Vazyme, China), while small fragments (e.g., His-tag) were generated via annealing of chemically synthesized primers. Primers for PCR molecular cloning or annealing were commercially synthesized by Tsingke Biotechnology (Dataset S2). Restriction enzyme digestion of plasmids was performed using *Bbs*I-HF and *Bsa*I-HF (New England Biolabs, China) and ligation was done by T4 DNA ligase (New England Biolabs), following the manufacturer’s protocols. For plasmid construction and assembly, a molar ratio of 1:3 (insert-to-vector) was routinely applied. The parts were cloned into the level 0 vectors of MoClo Tool Kit (Kit #1000000044; Addgene).

*Escherichia coli* DH5α was used for the transformation and reproduction of constructed plasmids. *E. coli* cells were cultured in LB medium at 37 °C. The medium was supplemented as required with 15% (w/v) agar, 50 μg mL^−1^ spectinomycin, 100 μg mL^−1^ ampicillin, and 40 μg mL^−1^ X-gal.

### Nuclear transformation of *Nannochloropsis*

*Nannochloropsis* cells from early exponential growth stages were used for transformation by nuclear electroporation (Li et al. 2014). Briefly, algal cells were inoculated at an initial OD750 of 0.15 and cultured in flasks for 48 h to reach ~ 10^7^ cells mL^−1^. A 50 mL aliquot was harvested and washed four times with ice-cold 384 mM sorbitol solution. The resulting competent algal cells were then resuspended in 300 μL chilled sorbitol solution, followed by addition of 5 μL sheared salmon sperm DNA (10 mg mL^−1^) and 3 μg linearized plasmid. The cell-DNA mixture was transferred to a prechilled 2-mm gap electroporation cuvette and incubated on ice for 30 min. Electroporation was conducted using a Gene Pulser Xcell™ system (Biorad, Hercules, USA) with parameters set to 50 μF capacitance, 600 Ω resistance, and 2400 V. Post-pulse cells were kept on ice for 5 min, then transferred to F/2 liquid medium for 24–48 h recovery under dim light (< 5 μmol photons m^-2^ s^-1^). Transformants were selected on F/2 agar plates containing 3 μg mL^−1^ bleomycin or 500 μg mL^−1^ hygromycin B, followed by 3–4 weeks incubation at 23 °C with continuous illumination of 30–40 μmol photons m^-2^ s^-1^.

### NanoLuc activity assay of *Nannochloropsis* transformants

Single colonies of *N. oceanica* were picked from antibiotic plates and cultured in 96-well plates under 50 μmol photos m^−2^ s^−1^ for six days. Luciferase activity was quantified using the Nano-Glo® Luciferase Assay System (Promega, Madison, USA) according to the manual’s procedures. Briefly, the luciferase substrate was diluted in F/2 medium at a 1:10,000 volume ratio, mixed thoroughly, and kept on ice until use. A 50 μL aliquot of algal culture with equal OD750 was transferred to a white 96-well plate, followed by addition of 150 μL substrate-containing medium. After incubation at room temperature for 3–5 min, luminescence intensity was measured using a SpectraMax i3x Multi-Mode Microplate Reader (Molecular Devices, San Jose, USA), which was designated as NanoLuc activity.

### Reverse transcription-quantitative PCR and RNA-Seq analysis

Total RNA was isolated from algal samples using the TransZol Up Plus RNA Kit (TransGen Biotech, China) following the manufacturer’s protocol. RNA quality and concentration were assessed using a Nanodrop 2000 spectrophotometer (Thermo Fisher Scientific) and 1% agarose gel electrophoresis. Approximately 1 µg of total RNA was reverse-transcribed into cDNA using the TransScript® All-in-One First-Strand cDNA Synthesis SuperMix (TransGen Biotech). Quantitative PCR (qPCR) was performed with the PerfectStart™ Green qPCR SuperMix (TransGen Biotech) under the following thermal cycling conditions: 50 °C for 2 min, 95 °C for 10 min, followed by 40 cycles of 95 °C for 15 s, 60 °C for 1 min, and a final extension at 65 °C for 5 s and 95 °C for 50 s. Relative mRNA expression levels were calculated using the 2^−ΔΔCt^ method, normalized to the endogenous β-actin reference gene.

For RNA-seq analysis, 10 μg of total RNA from each sample was used for transcriptome library construction and the following sequencing on an Illumina NovaSeq 6000 sequencing system (Illumina, USA) by Majorbio Biotechnology Co., Ltd (Shanghai, China). The clean reads were aligned to the genome of N. oceanica IMET1 (http://nandesyn.single-cell.cn/download) using the software TopHat (version 2.0.4). The transcriptome data were deposited in the National Microbiology Data Center (https://nmdc.cn/) with the accession number NMDC10018895. The gene transcriptional abundance was expressed as fragments per kilobase million (FPKM).

### Protein extraction and immunoblotting

Algal cells (5–10 mL culture) were pelleted by centrifugation at 5,000 rpm for 5 min and washed twice with sterile water. The pellet was resuspended in 200 μL Lysis Buffer (containing 50 mM Tris–HCl (pH 7.4), 150 mM NaCl, 1% Triton X-100, 1% sodium deoxy-cholate, and 0.1% SDS) with 0.1 mL acid-washed glass beads and homogenized using a high-throughput tissue disruptor SCIENTZ-48 (30 Hz, 1 min) (Scientz Biotech, China). Lysates were centrifuged at 13,000 × g for 15 min (4 °C), and the supernatant (20 µg protein) was resolved by 12% SDS-PAGE, followed by staining with the Beyotime Coomassie Blue Fast Staining Solution (Shanghai, China). Protein concentration was determined using the Beyotime BCA Protein Assay Kit.

For immunoblotting, proteins were transferred to PVDF membranes (Sigma-Aldrich). The membranes were then blocked with 5% non-fat milk in TBST for 2 h at room temperature, followed by incubation overnight at 4 °C with primary antibodies (Thermo Fisher Scientific) diluted in 5% milk-TBST. The antibodies included GFP Monoclonal Antibody (GF28R, HRP-conjugated), 6 × His Tag Monoclonal Antibody (HIS.H8, HRP-conjugated), HA Tag Monoclonal Antibody (2–2.2.14, HRP-conjugated), Flag Tag Monoclonal Antibody (FG4R, HRP-conjugated), c-Myc Monoclonal Antibody (9E10, HRP-conjugated), and Strep-Tag II Monoclonal Antibody (8C12). The former four antibodies were purchased from Thermo Fisher Scientific, while the latter one was from Abbkine (China). After four times of washing with 20 mM tris buffered saline with 0.1% Tween-20 (TBST) (10 min each), membranes were incubated with Anti-Mouse HRP-conjugated secondary antibody (1 h, room temperature) and washed again. Protein bands were visualized using an enhanced chemiluminescence detection system.

### Fluorescent microscopy observation

Positive transformants with fluorescent protein-coding genes in exponential growth stages were observed and imaged using an OLYMPUS laser-scanning confocal microscope (FV1000; OLYMPUS,Tokyo, Japan) and a fluorescence microscope (BX53; OLYMPUS). The fluorescence was detected as followed: eGFP (excitation at 488 nm, emission at 505 nm), YFP (excitation at 514 nm, emission at 527 nm), StayGold (excitation at 495 nm, emission at 505 nm), and chloroplast autofluorescence (excitation at 488 nm, emission at 650–750 nm).

### Pigment extraction and analysis

Algal samples were homogenized with 0.4 mm acid-washed glass beads in 1 mL chloroform:methanol (2:1, v/v; containing 0.05% butylated hydroxytoluene) using a high-throughput tissue disruptor SCIENTZ-48 (30 Hz, 1 min) (Scientz Biotech). After centrifugation, the organic phase was collected and the pellet was re-extracted until becoming colorless. A 0.75% NaCl solution (0.75 × methanol volume) was added for phase separation. The mixture was vortexed for 2 min, centrifuged at 2,000 × g (4 °C, 5 min), and the lower lower organic phase was transferred to amber vials and dried by nitrogen stream. Pigments were resuspended in acetone (100 μL per 1 mg biomass), filtered through a 0.22 μm Millipore organic membrane, and 10 μL was analyzed by a High Performance Liquid Chromatography (HPLC) system (LC-2050C, Shimadzu, Japan) equipped with a C18 column (4.6 × 250 mm; Osaka Soda, Japan). The elution rate was 1.0 mL min^−1^ with a linear gradient from100% solvent A (acetonitrile/methanol/H_2_O, 84:2:14; by vol) to 100% solvent B (methanol/ethyl acetate, 68:32; v/v) over a 15 min period, followed by 10 min of solvent B. Pigments were identified and quantified by using commercially standards.

### Isolation of thylakoid membranes

The canthaxanthin-rich algal cells (2 L) in the exponential growth phase were harvested for thylakoid membrane isolation and sucrose density gradient ultracentrifugation, following our previously described procedures (Liu et al. 2024a). Briefly, after pelleting the algal cells (5,000 × *g* for 5 min, 4 °C), they were washed three times with 50 mM HEPES buffer and resuspended in the same buffer. The resuspended cells were disrupted using a high-pressure homogenizer (AH-D150, ATS Engineering Limited, China) with three cycles at 600 Bar. The unbroken cells were removed by centrifugation at 2,000 × *g* for 10 min, and the thylakoids in the supernatant were collected by centrifugation at 150,000 × *g* for 1 h. The thylakoid membranes were then solubilized with 1.2% (w/v) dodecyl-α-D-maltopyranoside (α-DDM) at 0.5 mg Chlorophyll a/mL for 25 min at 4 °C. The resulting mixture was loaded onto a fresh 0.1–1.1 M linear sucrose density gradient containing 0.02% α-DDM. Ultracentrifugation was carried out for 18 h (150,700 × *g*, 4 °C) using a SW 40 Ti swing-out rotor (Beckman Coulter, USA) to separate fractions of thylakoid membranes.

### Statistical analysis

All the experiments were conducted with no less than three biological replicates, and the data were expressed as mean ± SD. Statistical analysis was performed according to Student’s *t*-test or one-way analysis of variance (ANOVA).

## Conclusion

The development of a Modular Cloning (MoClo) toolkit for *Nannochloropsis* marks a significant advancement in its synthetic biology applications. By incorporating 91 domesticated genetic parts and validating their functionality, this toolkit facilitates efficient gene assembly, transformant selection, and subcellular localization. The successful modular assembly and functional of keto-carotenoid biosynthesis pathways, resulting in the production of canthaxanthin and astaxanthin, demonstrates its potential for CO_2_-driven biotechnological applications. This toolkit enhances the flexibility and expansibility of *Nannochloropsis*-based synthetic biology systems, providing a critical platform for green and sustainable bio-production.

## Supplementary Information

Supplementary Material 1: Dataset S1. Primers used in this study. Dataset S2. Information about the gene parts shown in Fig. 1; Fig. S1. Schematic illustration of modular assembly from parts to modules and devices. Fig. S2. Fluorescence microscopy observation of subcellular localization of fluorescent proteins in *N. oceanica* transformant with pNC2-13 or pNC2-14. Fig. S3. The original immunoblotting pictures shown in Fig. 5. Fig. S4. HPLC chromatograph of carotenoid extracts from pNC2-18 or pNC2-19 transformants under NL or HL conditions. Fig. S5. Transcriptomic analysis between WT and canthaxanthin-rich or astaxanthin-rich strain of *Nannochloropsis*. Fig. S6. Quantification of canthaxanthin in wild type *Nannochloropsis gaditana* and the transformant with pNC2-18 under favorable growth conditions. Table S1. Pigment profiles of thylakoid membranes and the ultracentrifuged fractions of canthaxanthin-rich engineered *Nannochloropsis* strain.

## Data Availability

Data supporting this work are included in this published article and its supporting information files.
