# Supplementary material for: A modular synthetic biology toolkit unlocks metabolic engineering of the industrially relevant alga Nannochloropsis
Source: Adv Biotechnol (Singap). 2026 Feb 3;4(1):2. doi: 10.1007/s44307-026-00096-w (PMC12868398; doi:10.1007/s44307-026-00096-w)
Supplement: Supplementary file 1 — Supplementary Material 1: Dataset S1. Primers used in this study. Dataset S2. Information about the gene parts shown in Fig. 1; Fig. S1. Schematic illustration of modular assembly from parts to modules and devices. Fig. S2. Fluorescence microscopy observation of subcellular localization of fluorescent proteins in N. oceanica transformant with pNC2-13 or pNC2-14. Fig. S3. The original immunoblotting pictures shown in Fig. 5. Fig. S4. HPLC chromatograph of carotenoid extracts from pNC2-18 or pNC2-19 transformants under NL or HL conditions. Fig. S5. Transcriptomic analysis between WT and canthaxanthin-rich or astaxanthin-rich strain of Nannochloropsis. Fig. S6. Quantification of canthaxanthin in wild type Nannochloropsis gaditana and the transformant with pNC2-18 under favorable growth conditions. Table S1. Pigment profiles of thylakoid membranes and the ultracentrifuged fractions of canthaxanthin-rich engineered Nannochloropsis strain. [file 44307_2026_96_MOESM1_ESM.pdf]

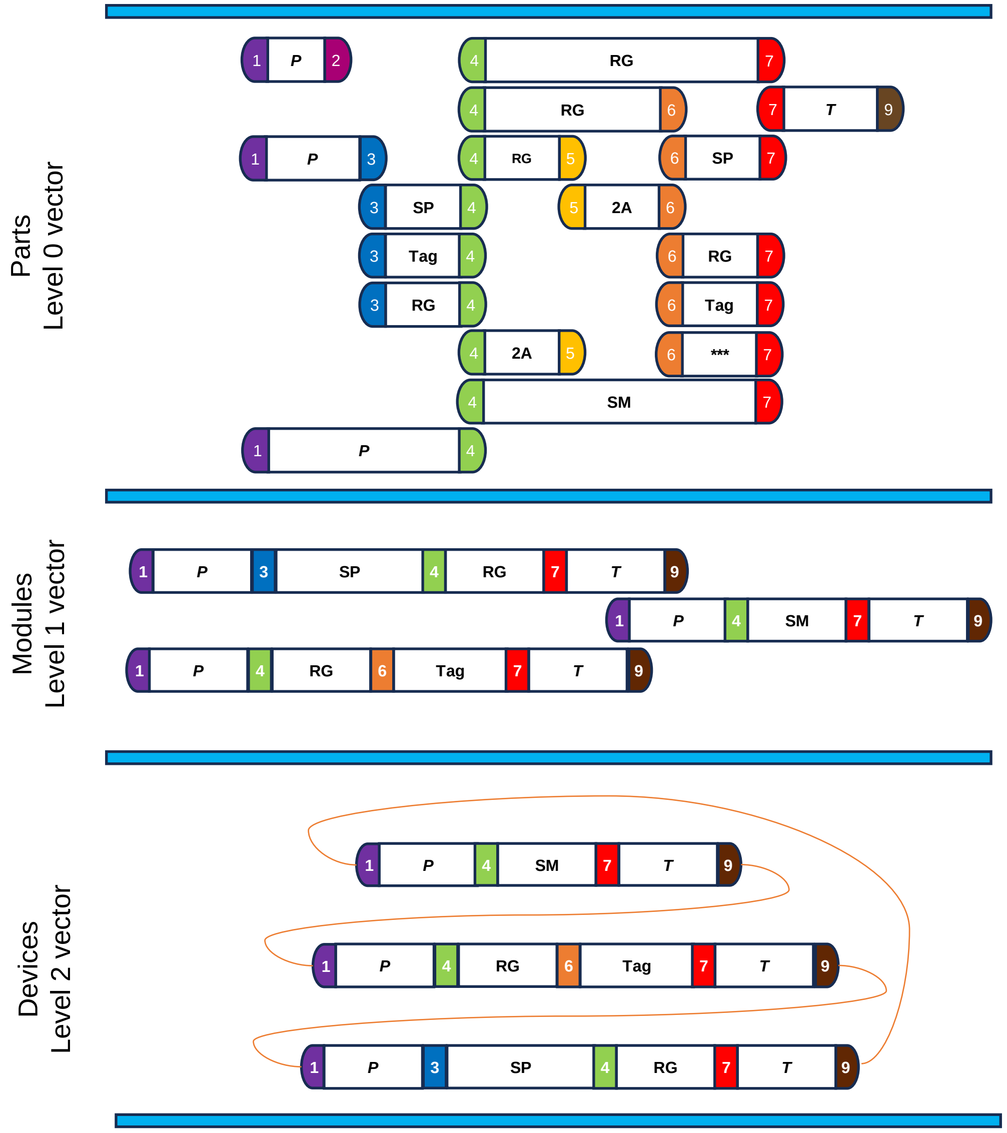

Fig. S1 Schematic illustration of modular assembly from parts to modules and devices.

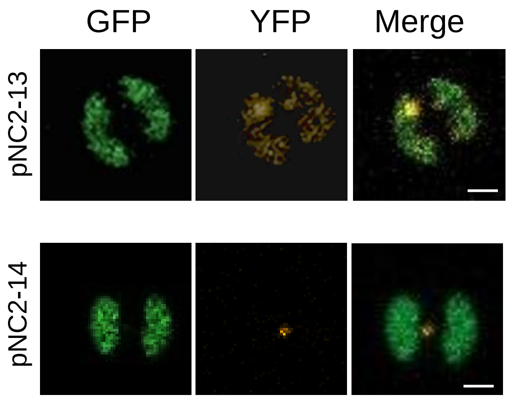

Fig. S2 Fluorescence microscopy observation of subcellular localization of fluorescent proteins in *N. oceanica* transformant with pNC2-13 or pNC2-14. Only GFP, YFP and the merge channels were shown. The transformants were the same ones shown in Figure 4. Scale bar: 2 µm.

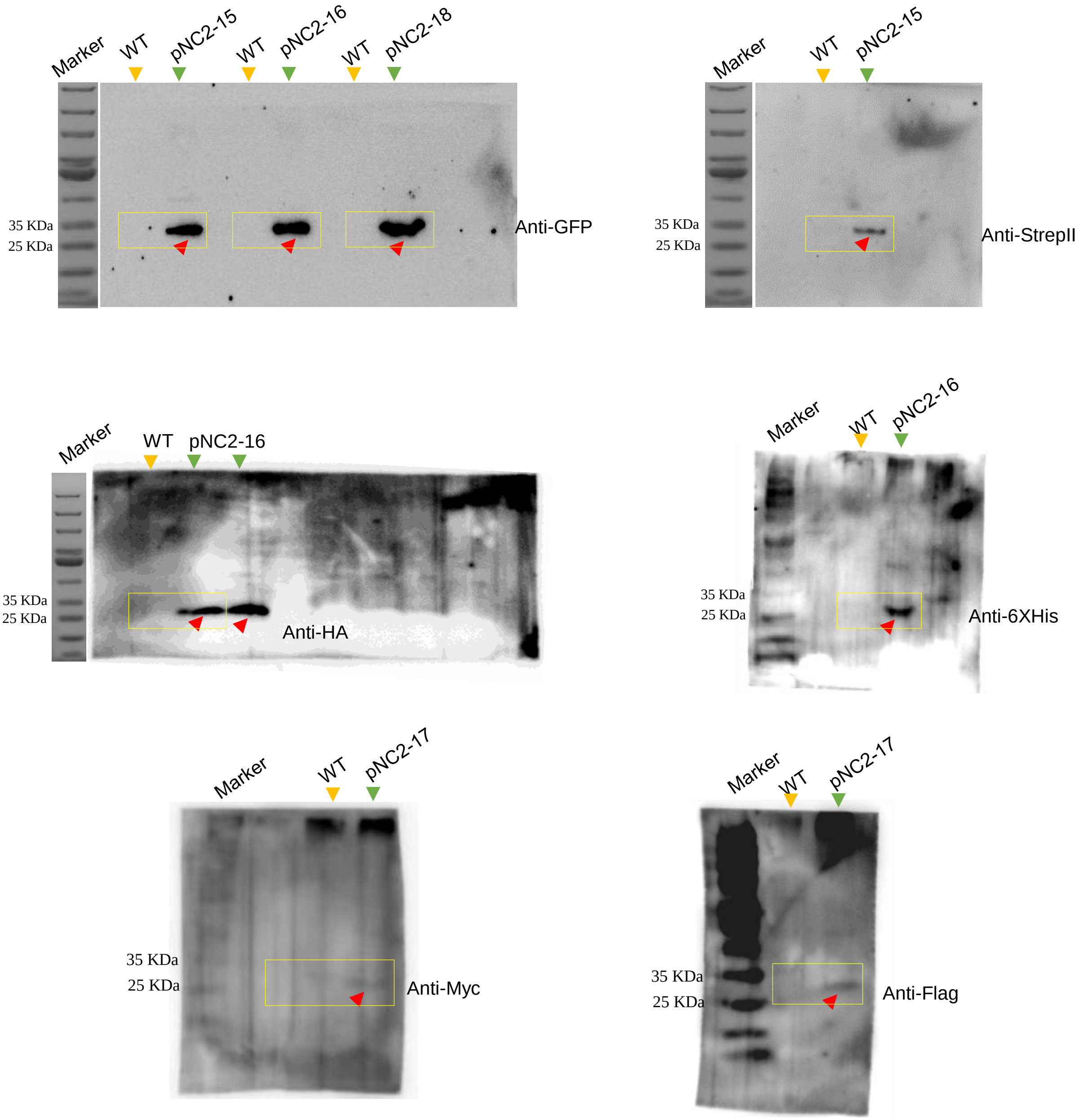

Fig. S3 The original immunoblotting pictures shown in Figure 5. For some blots, the protein marker was not loaded and a separated SDS-PAGE gel showing the marker was provided. The yellow boxes designate the cropped area shown in Figure 5.

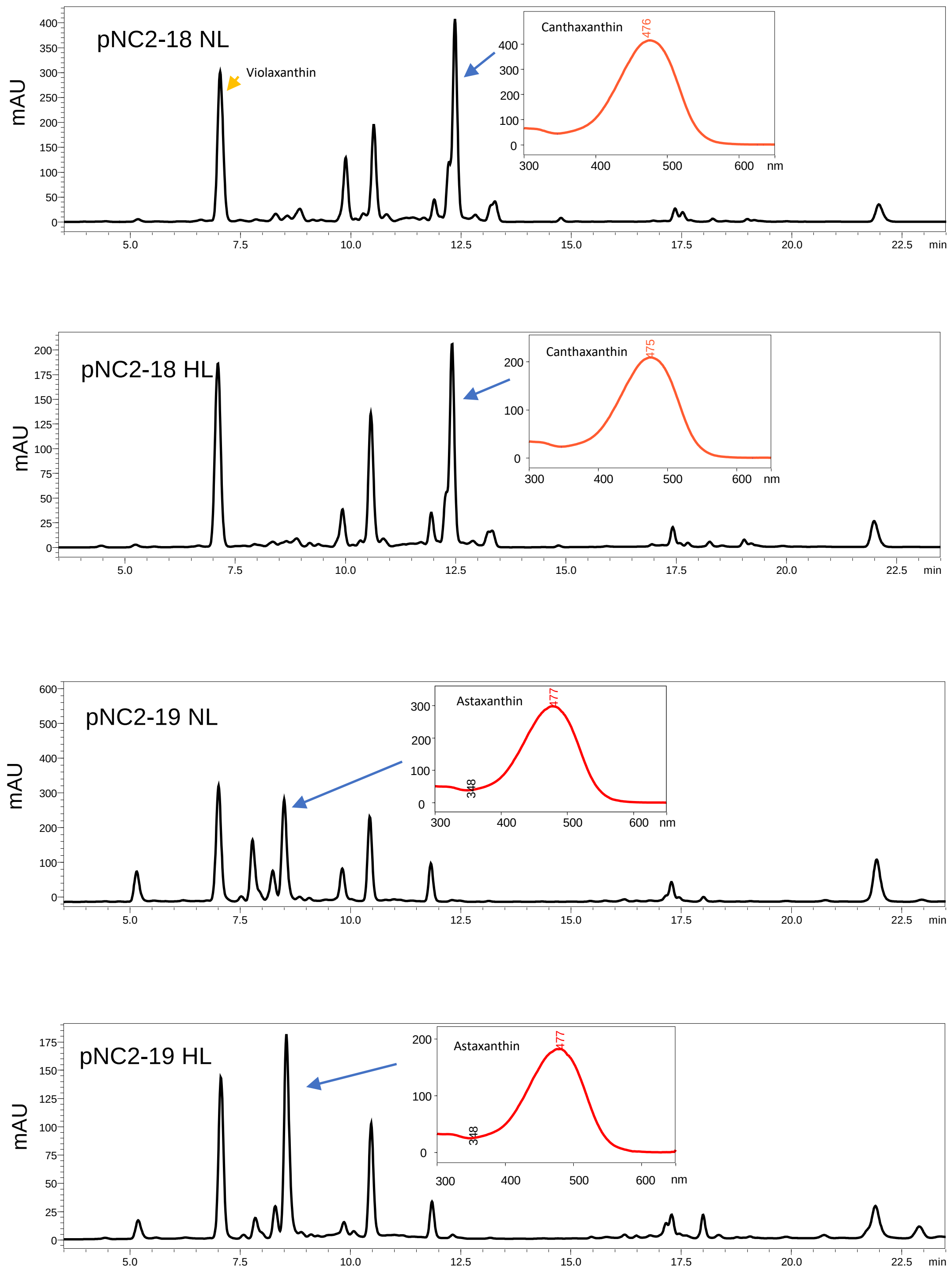

Fig. S4 HPLC chromatograph of carotenoid extracts from pNC2-18 or pNC2-19 transformants under NL or HL conditions. Absorption spectrum of canthaxanthin or astaxanthin was shown.

(a)

	Ast6h1	Ast6h2	WT6h1	WT6h2	Ast24h1	Ast24h2	WT24h1	WT24h2
Ast6h1	1	0.9957	0.9433	0.9434	0.8216	0.8182	0.8174	0.8175
Ast6h2	0.9957	1	0.9351	0.9371	0.8227	0.8258	0.8089	0.8165
WT6h1	0.9433	0.9351	1	0.9988	0.6351	0.6277	0.6929	0.69
WT6h2	0.9434	0.9371	0.9988	1	0.6383	0.6312	0.694	0.6926
Ast24h1	0.8216	0.8227	0.6351	0.6383	1	0.993	0.9431	0.946
Ast24h2	0.8182	0.8258	0.6277	0.6312	0.993	1	0.9205	0.9296
WT24h1	0.8174	0.8089	0.6929	0.694	0.9431	0.9205	1	0.9945
WT24h2	0.8175	0.8165	0.69	0.6926	0.946	0.9296	0.9945	1

(b)

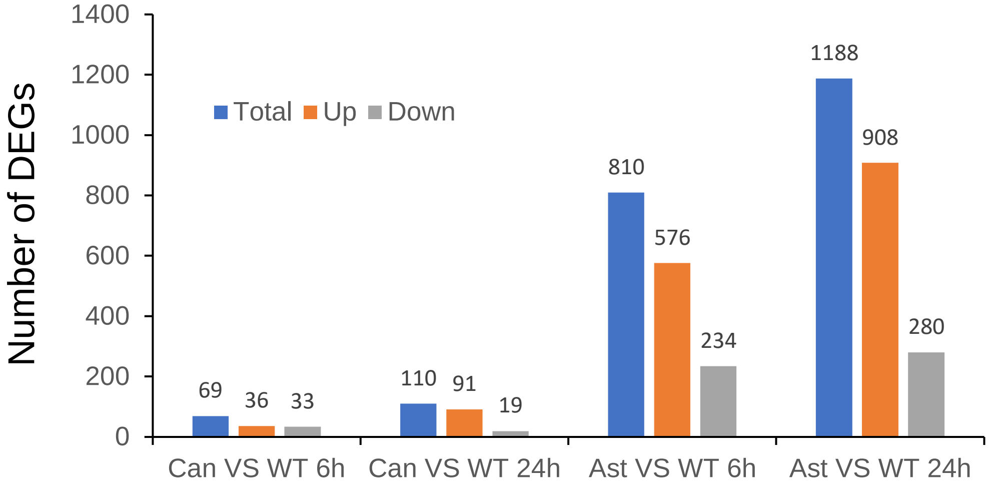

Fig. S5 Transcriptomic analysis between WT and canthaxanthin-rich or astaxanthin-rich strain of *Nannochloropsis*. (a) Pearson correlation of transcriptomes. (b) Number of differentially expressed genes (DEGs).

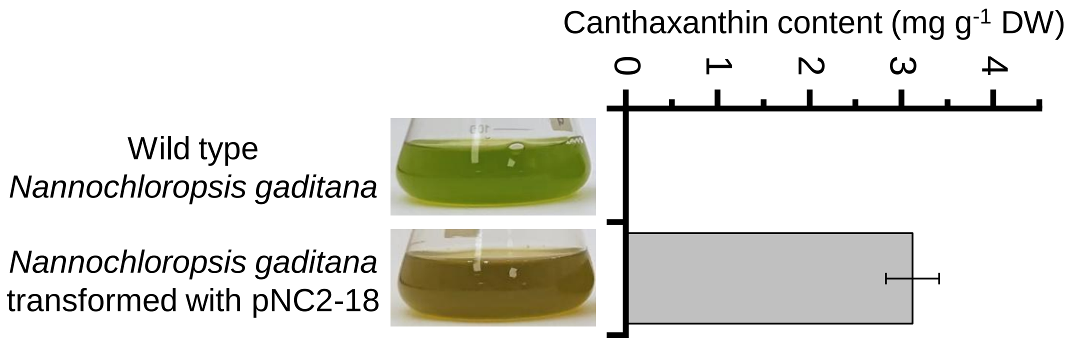

Fig. S6 Quantification of canthaxanthin in wild type *Nannochloropsis gaditana* and the transformant with pNC2-18 under favorable growth conditions

Table S1 Pigment profiles of thylakoid membranes and the ultracentrifuged fractions of canthaxanthin-rich engineered *Nannochloropsis* strain

	Violaxanthin	Vaucheriaxanthin	Vaucheriaxanthin ester	β-carotene	Canthaxanthin
TM	16.4 ± 0.8	6.1 ± 0.1	5.5 ± 0.4	3.8 ± 0.1	4.4 ± 0.2
VCP	33.4 ± 0.8	11.5 ± 0.3	7.1 ± 0.2	2.1 ± 0.1	6.6 ± 0.3
PSI-LHC	8.3 ± 0.3	0.5 ± 0.0	0.7±0.1	5.5±0.2	3.4±0.2

Carotenoids are expressed as mol per 100 mol chlorophyll *a*. TM, Thylakoid membranes; VCP, violaxanthin/chlorophyll *a* binding protein; PSI-LHC, photosystem I–light harvesting complex. Values are presented as mean ± SD (n = 3).
